# Intraoperative Transmission of Brucellae

**DOI:** 10.3201/eid3112.251518

**Published:** 2025-12

**Authors:** Pablo Yagupsky

**Affiliations:** Soroka University Medical Center, Ben-Gurion University of the Negev, Beer-Sheva, Israel

**Keywords:** Brucella melitensis, human brucellosis, zoonoses, bacteria, transmission, nosocomial infection, surgical procedures, biosafety, Israel

**To the Editor:** In a recent article, I. Potparić et al. described transmission of *Brucella melitensis* to an orthopedic surgeon during the irrigation of a spinal infection (*1*). Although rare, transmission of *B. melitensis* from patients to the surgical staff has been reported (*1*–*5*).

A low inhaled infective dose (10^2^ bacteria) characterizes brucellae, and the organism may enter the human body through the respiratory tract and conjunctivae, representing the most common agents of laboratory-acquired infections (*2*). Because the concentration of brucellae in body fluids and tissues is low, unseeded biologic specimens are not considered to pose a substantial transmission hazard. However, the risk of contagion increases exponentially after incubation of bacteriologic media. Thus, cultures of presumptive *Brucella* species bacteria should be processed in biologic safety cabinets (*2*). Physicians who care for patients with brucellosis, however, are not deemed to be at an increased risk because person-to-person transmission of the disease is extremely uncommon (*3*,*4*).

The reported events of intraoperative acquisition of the disease show common factors. The possibility of brucellosis in the patient was not suspected or contemplated, even when the patient was from an endemic region (*1*–*4*); the cases involved high-risk procedures, such as unprotected bone drilling and irrigation, or aspiration of respiratory secretions (*1*,*3*,*4*); the surgical procedure created aerosol clouds, or massive spillage of blood, amniotic fluid, or both (*3*,*4*); or the medical staff did not consistently wear face masks or goggles (*2*).

Of note, the 5 published events occurred in nonendemic countries that have advanced medical and laboratory diagnostic capabilities. In contrast, accounts from developing countries, where the zoonosis is rampant, are absent. We presume that lack of adequate epidemiologic surveillance and reporting, and the assumption that the disease was acquired outside the hospital, could be responsible for the missing reports.
